# Breath holding endurance: stability over time and relationship with self-assessed persistence

**DOI:** 10.1016/j.heliyon.2017.e00398

**Published:** 2017-09-06

**Authors:** Daisy G.Y. Thompson-Lake, Richard De La Garza II, Peter Hajek

**Affiliations:** a Wolfson Institute of Preventative Medicine, Queen Mary University of London, England, UK; b Menninger Department of Psychiatry and Behavioral Sciences, Baylor College of Medicine, Houston, TX

**Keywords:** Psychology

## Abstract

Breath holding (BH) endurance has been suggested as a measure of the distress tolerance that could predict the outcome of attempts to implement behavior changes, such as stopping smoking or illicit substance use. It is not known however, to what degree BH endurance is a variable trait that may vary depending on situational context, or a stable state characteristic. We measured BH in two groups of participants at baseline and 22 and 89 days (N = 62 and N = 41) post-baseline and in a third group at multiple times points across a 5-week period (N = 44). Participants also filled out a questionnaire created to assess their perceived persistence compared to peers. Correlations were found between baseline and final BH measures (*r*’s > 0.67, *p*’s < 0.0001) at all time points. When groups were combined, regardless of time point, Spearman’s rank correlation showed a strong positive correlation (r_s_ = 0.66, *p* < 0.0001). Self-assessed persistence was not related to BH endurance. This study provides evidence of the stability of BH across time when tested under the same conditions in young adults. Further research is needed to clarify whether BH is linked to behavioral outcomes.

## 1 Introduction

When people are asked to hold their breath for as long as they can, strong individual differences emerge. The average length of time people maintain breath-holding (BH) is one minute, which is not long enough for the build-up of carbon dioxide in blood to reach toxic levels [1]. However, the degree to which people can tolerate the subjective discomfort of BH varies greatly across individuals.

Based on an observation that smokers who showed extremely short BH endurance were unable to achieve smoking cessation [2]; and that immediate relapsers (<24 h abstinence) to cigarette smoking showed significantly shorter BH times than delayed relapsers (3 months or more abstinence) [3] it was suggested that BH could be a marker of a general ability to withstand self-imposed discomfort. These observations gave rise to the interest in ‘distress tolerance’ as a possible predictor of attempts to implement behavior changes that are accompanied by discomfort, such as stopping cigarette or substance use [4, 5].

Studies of the distress tolerance construct often include BH [6, 7, 8] as the test is quick and easy to administer. However, the results of studies examining the relationship between BH and behavior change have been mixed. Some studies found BH related to behavior change outcomes [3, 9] while others did not [10, 11].

This inconsistency of BH endurance to predict behavior change may stem from the two variables having only a tenuous link, but it is also possible that an individual’s BH endurance may vary depending on situational context. For instance, in healthy controls and in patients with schizophrenia, lower BH was related to greater self-reported negative affect [10]. BH has also been found to be significantly shorter during cigarette abstinence compared to smoking as usual [6], and shorter BH times were observed in smokers using placebo patch versus smokers using nicotine patch [12].

If momentary influences play a major role in BH endurance, BH is unlikely to be a reliable marker of a general personality trait. It is however also possible that the momentary effects are small and have little influence on where individual’s performance sits compared to others. Therefore, even if there is some fluctuation in BH over time, individual differences in BH could still remain stable.

BH has a high test-retest reliability when recorded within one session [6, 13, 14], but limited data exist on its stability over longer periods of time. We are aware of only one study that measured BH across a larger time-span. It included BH measures, taken after complete expiration, from 34 first-year psychology students one year apart. The test-retest correlation was *r* = 0.67 (*p* < 0.001) [15].

The current study further examined the stability of individual difference in BH over time by recruiting a larger sample of participants and measuring BH at multiple time points. Hypothesizing that when tested under stable conditions participants would not show a difference in BH endurance across time. In addition, we asked participants to estimate their perseverance compared to their peers to determine if subjective appraisal of general endurance correlates with BH.

## 2 Methods

### 2.1 Design

Second and third year medical students were recruited from The Barts and The London School of Medicine and Dentistry to obtain two separate student samples for a test-retest experiment (Group 1 and Group 2). In addition to this, a mixture of staff and students was recruited to provide BH at weekly intervals (Group 3). Altogether, 62 participants provided BH data 22 days apart in Group 1; 41 participants provided BH data 89 days apart in Group 2; and 44 participants provided weekly BH measures over five weeks in Group 3. Sample characteristics together with BH data are shown in Table 1. Informed consent was obtained from all individual participants included in the study. The experiments described in this study were approved by the human ethics review board at Queen Mary University of London. All procedures were in accordance with the ethical standards of the institutional and with the 1964Helsinki declaration and its later amendments.

**Table 1:** Demographics and BH results.

Group	N	Age (years) Mean (SD)	Sex (% female)	Baseline BH Mean (SD) (Seconds)	Final BH Mean (SD) (Seconds)	r
1: 22 days	62	21.2 (2.8)	61.3	53.45 (22.98)	58.13 (24.30)	0.67, *p* < 0.0001
2: 89 days	41	22.96 (3.8)^1^	68.3	60.38 (20.66)	59.85 (25.06)	0.71, *p* < 0.0001
3: 35 days	44	28.8 (8.7)^2^	64.5	57.27 (26.30)	64.20 (27.74)	0.78, *p* < 0.0001
All combined	133	23.7 (6.3)^3^	61.7	57.27 (24.39)	60.63 (25.84)	0.73, *p* < 0.0001

1 Due to missing data N = 37.

2 Due to missing data N = 35.

3 Due to missing data N = 120.

## 3 Materials and methods

### 3.1 Breath holding

BH was measured using the approach applied in Sutterlin et al. [15]. Participants were seated in a large auditorium with a stop clock projected onto screens at the front. Participants were asked, when instructed to do so, to close their eyes, take a deep breath in, and hold their breath for as long as possible until they were unable to hold it any longer. When participants could no longer hold their breath they breathed out and opened their eyes simultaneously, recording the time on the projected stop clock. BH was capped at 2 minutes (this BH duration was achieved by only one participant).

### 3.2 Persistence questionnaire (PQ)

A short questionnaire was developed to elicit assessment of perceived general persistence. The questionnaire consisted of 3 items relating to ‘willpower’, ‘persisting with difficult tasks’ and ‘giving in to temptations’ scored on a Likert scale of 1–5 (1 = “much more than average” to 5 = “much less than average”) (Appendix 1). Items were scored positively except ‘giving in to temptations’ which was scored negatively.

Group 1 provided measures at baseline and again in the same circumstances and setting 22 days later. Group 2 had 89 days break between the baseline and retest BH. Group 3 provided BH data once weekly for 5 weeks (35 days between the first and last measure). In Group 3, where availability and proximity permitted, participants were observed holding their breath. When this was not possible, participants provided their BH data via text or email.

### 3.3 Analysis

Bivariate analysis was conducted with BH and each individual item in the PQ, Sidak correction for multiple comparisons was applied to the significance level. PQ was also evaluated for internal consistency using Cronbach’s alpha. For calculating correlations with BH, baseline PQ results were correlated with baseline BH; and final PQ results were correlated with final BH.

In groups 1 and 2, paired t-tests were used to compare baseline and final BH. In group 3, a one way repeated measures analysis of variance (RMANOVA) was conducted to evaluate the effect of time on duration of BH at each weekly measurement. Post-hoc t-tests were carried out between each sequential BH (i.e. Baseline BH vs. BH2, BH2 vs. BH3 etc.) as well as between the baseline and final BH. Linear regressions were carried out between baseline BH and each weekly BH. All significance levels were corrected for multiple comparisons. T-tests (for absolute values) and Spearman’s rank correlation (for ranked values) were carried out for each BH compared with baseline.

Finally, all groups were combined. Fourteen participants from group 2 (89 days) who were also re-enrolled for group 3 (35 day) were removed. Correlations of baseline BH versus final BH were carried out. In addition, a Spearman’s test that measures the strength and direction of association between two ranked variables was used to assess the correlation between rank-order of the BH measures at the two time points. All analyses were conducted using SPSS v.23.

## 4 Results

No correlations were found between BH endurance and the PQ composite score or any of the three PQ items in any group (all *p*’s > 0.14).

The PQ had acceptable internal consistency (α = 0.66). In group 1, a significant correlation was found between the two BH measures (Table 1). BH times were not significantly different between baseline BH versus final BH (*t =* -1.9, *p =* 0.06).

In group 2, a significant correlation was found between the two BH measures (Table 1). BH times were not significantly different between baseline BH versus final BH (*t =* −1.9, *p =* 0.85). In group 3 significant correlations were also found between the baseline and the final BH (Table 1). BH times were significantly different between baseline BH versus final BH (*t =* −2.6, *p =* 0.01). When all subjects were combined (N = 133) significant correlations were found between baseline BH versus final BH (Table 1).

In group 3, significant strong correlations were found between each sequential BH measure. Baseline BH vs. BH2 (*r* = 0.88, *p* < 0.0001), BH2 vs. BH3 (*r* = 0.89, *p* < 0.0001), BH3 vs. BH4 (*r* = 0.90, *p* < 0.0001), and BH4 vs. BH5 (*r* = 0.90, *p* < 0.0001). Assumptions of RMANOVA were met, with the exception of Mauchly's Test of Sphericity therefore a Greenhouse-Geisser correction was used which showed mean BH differed significantly between time points [F(3.227, 138.76) = 2.835, p = 0.037]. However, after Sidak correction for multiple comparisons post-hoc analysis revealed no significant effect of time on BH endurance. (*ps >* .125) (Table 2). All rank order correlations of weekly BH with baseline showed strong correlations (all *p*’s < 0.001) (Table 2). When all subjects were combined (N = 133) Spearman’s rank correlation between baseline and final BH across the three samples was r_s_ = 0.67, *p* < 0.0001.

**Table 2:** Changes in weekly BH from baseline (N = 44).

	Baseline	BH2	BH3	BH4	BH5
BH (secs) Mean (SD)	57.3 (26.3)	61.5 (28.7)	63.9 (26.4)	61.8 (26.5)	64.2 (27.7)
Difference from baseline (secs)	–	4.2	6.6	4.5	6.9
*P*	–	*0.35 (ns)*	*0.11 (ns)*	*0.45 (ns)*	*0.12 (ns)*
r_s_ (all p’s ≪0.001)	–	0.84	0.80	0.84	0.73

## 5 Discussion

In a sample of young adults, BH endurance was significantly stable at multiple time points. This applied to the mean BH as well as to the relative positions of individuals compared to their peers. This opens a possibility that BH may be related to other personality characteristics. However, BH was not related to a brief questionnaire eliciting subjective perception of relative perseverance and endurance.

Our key result regarding the test-retest reliability of BH supports previous finding [15]. The test-re-test correlations in our study ranged between r = 0.66 and r = 0.90 while the previous study that tested the participants one year apart reported a test-retest correlation of r = 0.67. Pearson’s correlation of r = 0.70 and above is considered an acceptable marker of stability [16, 17] and such correlations are deemed high for a single item measure [18, 19] and suggest a reasonable stability over time. We observed some changes in BH over a 5 week period, but these were only modest (a 5% improvement in average BH endurance overall), and after correction for multiple comparisons, not significant. The rank-order correlation shows that on relative performance; participants maintained their relative position to their peers over time.

The questionnaire measure of self-assessed relative persistence (PQ) was not related to BH. BH endurance may not reflect personality traits that the PQ was trying to assess or the questionnaire may not be assessing the relevant traits adequately. The scale revealed only a moderate reliability, although this may be a result of a low number of factors included in the analysis [20, 21]. An alternative explanation for the poor correlations between the assessment and the BH could be that the measure relies on subjective ratings of one’s persistence compared to other people and individuals may not be very good at such estimates [22]. Overall the finding of no correlation between PQ and BH tallies with previous research which found no links between BH and executive function tasks typically used to assess self-regulatory traits [15].

The study has several limitations. The sample comprised healthy young people. In older samples where there may be changes in lung function over time, BH may be less stable. Practical considerations precluded use of more measures of distress tolerance and self-regulation. The lecture hall settings may have led to some spurious results in participants who did not comply with instructions, but this would be likely to make our finding conservative. In this context, it is reassuring that the mean BH after inspiration of our sample was close to the general population norm of one minute [1].

Demonstrating that BH is a stable characteristic over time has important implications for future studies examining its relationship to other behaviors and outcomes, such as its value in predicting the outcomes of attempts at difficult behavior changes.

## Declarations

### Author contribution statement

Daisy Thompson-Lake: Conceived and designed the experiments; Performed the experiments; Analyzed and interpreted the data; Contributed reagents, materials, analysis tools or data; Wrote the paper.

Richard De La Garza, II: Analyzed and interpreted the data.

Peter Hajek: Conceived and designed the experiments.

### Funding statement

This work was supported by UKCTAS (grant number MR/K023195/1)

### Competing interest statement

The authors declare no conflict of interest.

### Additional information

Supplementary content related to this article has been published online at http://dx.doi.org/10.1016/j.heliyon.2017.e00398.

No additional information is available for this paper.
