# Supplementary material for: Breath holding endurance: stability over time and relationship with self-assessed persistence
Source: Heliyon. 2017 Sep 6;3(9):e00398. doi: 10.1016/j.heliyon.2017.e00398 (PMC5591393; doi:10.1016/j.heliyon.2017.e00398)
Supplement: Appendix 1 [file mmc1.docx]

Appendix 1

Persistence Questionnaire

How do you think you compare with other people you know in terms of the following (please tick the box which best characterizes you)

|  | Much more than average | Above Average | About Average | Less than Average | Much Less than Average |
| --- | --- | --- | --- | --- | --- |
| Willpower |  |  |  |  |  |
| Persisting on difficult tasks |  |  |  |  |  |
| Giving in to temptations |  |  |  |  |  |
